# Initial Alignment of Large Azimuth Misalignment Angles in SINS Based on Adaptive UPF

**DOI:** 10.3390/s150921807

**Published:** 2015-08-31

**Authors:** Jin Sun, Xiao-Su Xu, Yi-Ting Liu, Tao Zhang, Yao Li

**Affiliations:** Key Laboratory of Micro-Inertial Instrument and Advanced Navigation Technology, Ministry of Education, School of Instrument Science and Engineering, Southeast University, Nanjing 210096, China; E-Mails: sunjin8607986@126.com (J.S.); gcdlyt1985@163.com (Y.-T.L.); ztandyy@163.com (T.Z.); lyjenny11@163.com (Y.L.)

**Keywords:** strapdown inertial navigation system (SINS), large misalignment angle, initial alignment, unscented particle filter (UPF), adaptive UPF

## Abstract

The case of large azimuth misalignment angles in a strapdown inertial navigation system (SINS) is analyzed, and a method of using the adaptive UPF for the initial alignment is proposed. The filter is based on the idea of a strong tracking filter; through the introduction of the attenuation memory factor to effectively enhance the corrections of the current information residual error on the system, it reduces the influence on the system due to the system simplification, and the uncertainty of noise statistical properties to a certain extent; meanwhile, the UPF particle degradation phenomenon is better overcome. Finally, two kinds of non-linear filters, UPF and adaptive UPF, are adopted in the initial alignment of large azimuth misalignment angles in SINS, and the filtering effects of the two kinds of nonlinear filter on the initial alignment were compared by simulation and turntable experiments. The simulation and turntable experiment results show that the speed and precision of the initial alignment using adaptive UPF for a large azimuth misalignment angle in SINS under the circumstance that the statistical properties of the system noise are certain or not have been improved to some extent.

## 1. Introduction

The initial alignment is a key technology in SINS, and the alignment precision and the alignment time are two important indexes which affect the overall system performance. With the application field of navigation systems continuing to expand, most application environments cannot meet the condition that the initial misalignment angle is a large angle and the noise is a Gaussian white noise, so continuing to use the traditional linear navigation system model and Kalman Filter (KF) will produce a greater model error and estimation error, which make the navigation parameters unbelievable [1]. According to this situation, research is mainly divided into two aspects; one is the research on a nonlinear model of the inertial navigation system [2,3,4,5,6,7,8,9,10], the initial alignment is usually divided into two stages of coarse alignment and fine alignment, as the strict mathematical error model of the inertial navigation system is a set of nonlinear differential equations, there must be some modeling error when using a linear model to approximate a nonlinear model. The small misalignment angle linear model is satisfied only under the condition of assuming that the various error sources are minor. However, the actual initial misalignment angles are large in many cases and, therefore, directly adopting a nonlinear model can reflect more truly the error propagation characteristics. In [3], the initial alignment error model of an inertial navigation system is established with the use of a disturbance approximation method, in which the azimuth misalignment angle is large and the horizontal misalignment angles are small, however, the model limits that the horizontal misalignment angles are small, affecting its scope of application. Reference [4] unified two processes of coarse alignment and fine alignment, and the universal SINS initial alignment nonlinear error models are established by using three status to describe the Ψ angle; and in [5] a three misalignment angles non-linear error mode expressed by multiplicative quaternion is derived, where the derivation process is without linearization to ensure the accuracy of the model. The other is the study of non-linear filters [11,12,13]. In the commonly-used methods of nonlinear filtering, there are the extended Kalman Filter (EKF), unscented Kalman Filter (UKF), particle filter (PF), and UKF-PF (UPF). The most famous algorithm to solve the problem of nonlinear filtering is the extended Kalman Filter (EKF) [1]. This filter, based upon the principle of linearizing the process and observation models using Taylor series expansions, has been successfully implemented in some nonlinear problems. Since the high-order terms above the second-order term are discarded during the linear process, the EKF can only be suitable for the estimation of poor nonlinear objects, and the stronger the nonlinear characteristics of the estimated object are, the greater the estimation error is, which can even cause divergence. Unlike EKF, UKF is based on the covariance matrix of the estimation vector and the measurement vector to determine the optimum gain matrix, the covariance matrix is calculated based on the reproducible double sigma sample points, where these sample points are determined according to the system equation and the measurement equation. Therefore, during the process of calculating the optimum gain matrix, no additional conditions are imposed on the system equation and the measurement equation in UKF, so the algorithm is not only suitable for linear objects, but also for non-linear objects. However, UKF is an approximate form of linear minimum variance estimation, while the standard Kalman filter is a precise linear minimum variance estimation, so only under nonlinear conditions can UKF fully reflect its superiority. The conditional mean is calculated by PF directly according to the probability density, and the probability density is determined by the EKF or UKF approximately, the estimated value ${\hat{X}}_{k}$ at $K^{th}$ time is determined by the weighted average value of multitudinous sample values (particles) that have different distributions. EKF or UKF must be completed once when each particle is calculated, so PF is suitable for the condition that the system and measurement are nonlinear, and the estimated accuracy is higher than the accuracy of using EKF or UKF alone, but the calculation level is much higher than EKF and UKF and it can be invalid when the sensor is very accurate or the data experience abrupt changes. The core of PF is choosing a reasonably recommended probability density, so the closer the recommended density selected is to the true density, the better the filter effect is, otherwise it will be worse, or even divergent. If we combine PF with UKF, the recommended density is determined by UKF, which can not only solve the problem of the degradation of particles, but also enable particles to get the latest *a posteriori* information of the measurement vector when they update, which is helpful for particles to move toward the area with higher likelihood ratio. PF combined with UKF is called UPF. For the condition that the treated object is non-linear and the white noise does not obey the Gaussian distribution, the highest estimation accuracy is achieved by UPF, then PF, UKF follows, and EKF follows it again. Above all, to a certain extent, the use limitations of UPF are less, and the filtering result is better than that of the other kinds of algorithms.

This paper, based on the UPF filtering algorithm, aims at improving the deficiencies of UPF. An approach based on a covariance matching criterion is adopted to judge the convergence and divergence situation of the filter, the covariance of the prediction error is revised and the filter gain is adjusted by an approach of introducing an adaptive attenuation factor, then achieving the goal that restrains and eliminates the divergence phenomenon in the filter and further improves the filter capability of fast tracking. To some extent, it reduces the influence on the system due to system simplification, the uncertain statistical properties of the noise, meanwhile, better overcome the UPF particle degradation phenomenon.

The rest of this paper is organized as follows: the nonlinear error model of SINS based on Euler platform error angles is established in Section 2. Then the detailed adaptive UPF filtering algorithm is designed in Section 3. In Section 4, the factors that influence the adaptive UPF filter are analyzed. In Section 5, two kinds of filtering algorithms are used for the simulation experiment. In Section 6, a turntable experiment for the proposed method with a certain type of SINS is carried out by contrast with that of initial alignment of large azimuth misalignment angle in SINS based on UPF. Finally, conclusions are drawn in Section 7.

## 2. Nonlinear Error Model of SINS

For the case of SINS with a large misalignment angle, the error caused by the rotation order cannot be ignored and the error model of SINS must be re-established according to the large misalignment situation. Euler platform error angles are used to indicate the misalignment angle between the ideal navigation coordinate and calculated navigation coordinate, and the rotation order of the group error angles should be considered. The corresponding nonlinear error model of SINS is established.

This paper follows the coordinate system selection:

1. *i* frame—geocentric inertial coordinate, the origin is at the center of the Earth , the *x_i_* axis points at equinox, the *z_i_* axis is along the Earth’s axis of rotation, the *y_i_* axis and the *x_i_* axis, the *z_i_* axis constitute the right-handed coordinate system;
2. *e* frame—the Earth coordinate, the origin is at the center of the Earth, the *x_e_* axis passes through the intersection of the prime meridian and the equator, the *z_e_* axis passes through the North Pole of the Earth, and the *y_e_* axis passes through the intersection of the eastern longitude 90° meridian and the equator;
3. *n* frame—the navigation coordinate, here we select the “East-North-Up (ENU)” geographic coordinate system as the navigation coordinate;
4. *b* frame—“Right-Front-Up” coordinate for the SINS coordinate.

*n* frame has followed through three Euler angles rotation to *b* frame, the three Euler angles are denoted by the heading angle Ψ∈(−π  π], pitch angle θ∈[−π/2  π/2], and roll angle γ∈(−π  π], and the rotation transformation relationship between the *n* frame and *b* frame can be described by the attitude matrix $C_{b}^{n}$ [10,14].

### 2.1. Attitude Error Equation

In the actual navigation system there exist various disturbances and measurement errors, so a rotational error usually exists between the SINS calculation platform coordinate (*n'* frame) and the ideal navigation coordinate (*n* frame). The *n* frame requires one to rotate three angles successively in a certain order and then it can coincide with the *n'* frame, and now it is assumed that three rotations are successively rotated around the *z*-axis, *x*-axis, *y*-axis, and the turned angles denoted as $\varphi_{z}$, $\varphi_{x}$ and $\varphi_{y}$, so their vector expression form is $\varphi ={\left[\varphi_{x}\;\varphi_{y}\;\varphi_{z}\right]}^{T}$. Three rotations corresponding with the attitude transformation matrix follow as $C_{\varphi_{z}}$, $C_{\varphi_{x}}$ and $C_{\varphi_{y}}$, so the transformation matrix from the *n* frame to the *n'* frame can be expressed as:

(1) $$C_{n}^{n^{\prime }}=C_{\varphi_{y}}C_{\varphi_{x}}C_{\varphi_{z}}$$

where $C_{\varphi_{z}}=\left[\begin{array}{ccc} \cos\varphi_{z} & \sin\varphi_{z} & 0\\ -\sin\varphi_{z} & \cos\varphi_{z} & 0\\ 0 & 0 & 1 \end{array}\right]$, $C_{\varphi_{x}}=\left[\begin{array}{ccc} 1 & 0 & 0\\ 0 & \cos\varphi_{x} & \sin\varphi_{x}\\ 0 & -\sin\varphi_{x} & \cos\varphi_{x} \end{array}\right]$ and $C_{\varphi_{y}}=\left[\begin{array}{ccc} \cos\varphi_{y} & 0 & -\sin\varphi_{y}\\ 0 & 1 & 0\\ \sin\varphi_{y} & 0 & \cos\varphi_{y} \end{array}\right]$

If it is assumed that the angular velocity of the *n'* frame relative to the *n* frame is $\omega_{nn^{\prime }}^{n^{\prime }}$, the differential equation for the Euler platform error angles is:

(2) $$\dot{\varphi }=C_{\omega }^{-1}\omega_{nn^{\prime }}^{n^{\prime }}$$

where $C_{\omega }^{-1}=\frac{1}{\cos\varphi_{x}}\left[\begin{array}{ccc} \cos(\varphi_{y})\cos(\varphi_{x}) & 0 & \sin(\varphi_{y})\cos(\varphi_{x})\\ \sin(\varphi_{y})\sin(\varphi_{x}) & \cos(\varphi_{x}) & -\cos(\varphi_{y})\sin(\varphi_{x})\\ -\sin(\varphi_{y}) & 0 & \cos(\varphi_{y}) \end{array}\right]$

From [10] the SINS attitude error equation can be obtained as follows:

(3) $$\dot{\varphi }=C_{\omega }^{-1}[(I-C_{n}^{n^{\prime }}){\hat{\omega }}_{in}^{n}+C_{n}^{n^{\prime }}\delta \omega_{in}^{n}-C_{b}^{n^{\prime }}\delta \omega_{ib}^{b}]$$

### 2.2. Velocity Error Equation

In the navigation frame, the velocity differential equation of SINS is [10,14,15]:

(4) $${\dot{v}}^{n}=C_{b}^{n}f_{sf}^{b}-(2\omega_{ie}^{n}+\omega_{en}^{n})\times v^{n}+g^{n}$$

However, the velocity differential equation contains errors in the actual system, so now the SINS velocity differential equations should be as follows:

(5) $$\dot{\tilde{v}}=C_{b}^{n^{\prime }}{\tilde{f}}_{sf}^{b}-(2{\tilde{\omega }}_{ie}^{n}+{\tilde{\omega }}_{en}^{n})\times {\tilde{v}}^{n}+{\tilde{g}}^{n}$$

where ${\tilde{v}}^{n}=v^{n}+\delta v^{n}$, ${\tilde{f}}_{sf}^{b}=f_{sf}^{b}+\delta f_{sf}^{b}$, ${\tilde{\omega }}_{ie}^{n}=\omega_{ie}^{n}+\delta \omega_{ie}^{n}$, ${\tilde{\omega }}_{en}^{n}=\omega_{en}^{n}+\delta \omega_{en}^{n}$, ${\tilde{g}}^{n}=g^{n}+\delta g^{n}$, and $\delta f_{sf}^{b}$ is the accelerometer measurement error. The velocity error equation of SINS can be obtained directly by means of Equation (5) minus Equation (4):

(6) $$\begin{array}{l} \delta {\dot{v}}^{n}=[I-{\left(C_{n}^{n^{\prime }}\right)}^{T}]C_{b}^{n^{\prime }}{\tilde{f}}_{sf}^{b}+{\left(C_{n}^{n^{\prime }}\right)}^{T}C_{b}^{n^{\prime }}f_{sf}^{b}-(2\delta \omega_{ie}^{n}+\delta \omega_{en}^{n})\times {\tilde{v}}^{n}\\ \;-(2{\tilde{\omega }}_{ie}^{n}+{\tilde{\omega }}_{en}^{n})\times \delta v^{n}+(2\delta \omega_{ie}^{n}+\delta \omega_{en}^{n})\times \delta v^{n}+\delta g^{n} \end{array}$$

The calculation parameters error $\delta \omega_{ie}^{n}$ and $\delta \omega_{en}^{n}$ in Equations (3) and (6) can be specifically expressed as:

(7) $$\delta \omega_{ie}^{n}={\hat{\omega }}_{ie}^{n}-\omega_{ie}^{n}=\left[\begin{array}{c} 0\\ \omega_{ie}[\cos\tilde{L}-\cos(\tilde{L}-\delta L)]\\ \omega_{ie}[\sin\tilde{L}-\sin(\tilde{L}-\delta L)] \end{array}\right]$$

(8) $$\delta \omega_{en}^{n}={\tilde{\omega }}_{en}^{n}-\omega_{en}^{n}=\left[\begin{array}{c} -\delta L\\ \dot{\tilde{\lambda }}\cos\tilde{L}-(\dot{\tilde{\lambda }}-\delta \dot{\lambda })\cos(\tilde{L}-\delta L)\\ \dot{\tilde{\lambda }}\sin\tilde{L}-(\dot{\tilde{\lambda }}-\delta \dot{\lambda })\sin(\tilde{L}-\delta L) \end{array}\right]$$

where $\tilde{L}=L+\delta L$ and $\tilde{\lambda }=\lambda +\delta \lambda$.

### 2.3. Initial Alignment Error Model of Large Azimuth Misalignment Angle in SINS

It is assumed that the two horizontal misalignment angles are both small angles; assuming the gyro measurement errors $\delta \omega_{ib}^{b}$ are mainly composed of the constant drift error $\varepsilon^{b}$ and zero mean Gaussian white noise $w_{g}^{b}$, the accelerometer measurement error $\delta f_{sf}^{b}$ are mainly the constant bias error $\nabla^{b}$ and zero mean Gaussian white noise $w_{a}^{b}$, the gravity error term $\delta g^{n}$ is ignored, ${\tilde{v}}^{n}=\delta v^{n}$ holds under the static base, then the state equation of the initial alignment filtering model is obtained [10]:

(9) $$\{\begin{array}{l} {\dot{\varphi }}_{i}=C_{\omega }^{-1}[(I-C_{n}^{n^{\prime }}){\tilde{\omega }}_{in}^{n}+C_{n}^{n^{\prime }}\delta \omega_{in}^{n}-C_{b}^{n^{\prime }}\delta \omega_{ib}^{b}]\;(i=x,y,z)\\ \delta {\dot{v}}_{i}^{n}=[I-{\left(C_{n}^{n^{\prime }}\right)}^{T}]C_{b}^{n^{\prime }}{\tilde{f}}_{sf}^{b}+{\left(C_{n}^{n^{\prime }}\right)}^{T}C_{b}^{n^{\prime }}\nabla^{b}-(2{\tilde{\omega }}_{ie}^{n}+{\tilde{\omega }}_{en}^{n})\times \delta v^{n}+{\left(C_{n}^{n^{\prime }}\right)}^{T}C_{b}^{n^{\prime }}w_{a}^{b}\;(i=x,y,z)\\ {\dot{\varepsilon }}_{i}^{b}=0\;(i=x,y,z)\\ {\dot{\nabla }}_{i}^{b}=0\;(i=x,y,z) \end{array}$$

In this paper the state error vector can be expressed as $X={\left[\begin{array}{llllllllll} \varphi_{x} & \varphi_{y} & \varphi_{z} & \delta v_{x}^{n} & \delta v_{y}^{n} & \varepsilon_{x}^{b} & \varepsilon_{y}^{b} & \varepsilon_{z}^{b} & \nabla_{x}^{b} & \nabla_{y}^{b} \end{array}\right]}^{T}$, and the noise vector $W=\left[\begin{array}{cccccccccc} w_{gx}^{b} & w_{gy}^{b} & w_{gz}^{b} & w_{ax}^{b} & w_{ay}^{b} & 0 & 0 & 0 & 0 & 0 \end{array}\right]$. Establishing the filtering state model, and making the velocity error of SINS $Z=\delta v^{n}$ as the observation equation:

(10) $$\{\begin{array}{l} \dot{X}=f(X)+G(X)W\\ Z=HX+V \end{array}$$

The specific expressions of f(X) and G(X) can refer to Equation (9), and the matrix H is given as $H=\left[\begin{array}{ccc} 0_{2\times 3} & I_{2\times 2} & 0_{2\times 5} \end{array}\right]$, and V is the measurement noise.

## 3. UPF and Adaptive UPF

### 3.1. UPF Algorithm

Suppose the discrete form of the system equation and the observation equation is as follows:

(11) $$\{\begin{array}{l} X_{k}=f(X_{k-1},u_{k-1})+W_{k-1}\\ Z_{k}=h(X_{k})+V_{k} \end{array}$$

where $W_{k}$ and $V_{k}$ are the uncorrelated white Gaussian noise, whose mean value is zero and the variance matrix is $Q_{k}$ and $R_{k}$, then the specific steps of the UPF algorithm are as follows [13,14]:

1. Initialization: *k* = 0; Suppose the initial state variable $x_{0}\sim p(x_{0})$, the covariance matrix is $P_{0}$, we sample particles $\chi_{0}^{\left(i\right)}(i=1,2,3\ldots ,N)$ from the initial probability distribution $p(x_{0})$, for the simplified calculation, let $\chi_{0}^{\left(i\right)}\sim N({\bar{\chi }}_{0}^{\left(i\right)},P_{0}^{i})$, where ${\bar{\chi }}_{0}^{\left(i\right)}=\chi_{0}^{\left(i\right)}$ and $P_{0}^{i}=P_{0}$;
2. The forecast and sampling of the weighted particles: *k* = 1, 2, …; make use of the unscented Kalman filtering for particles to forecast, and calculate σ sampling points:
  (12) $$\chi_{(0)k-1}^{\left(i\right)}={\bar{\chi }}_{k-1}^{\left(i\right)}$$
  (13) $$\chi_{(j)k-1}^{\left(i\right)}={\bar{\chi }}_{k-1}^{\left(i\right)}+\gamma {\left(\sqrt{P_{k-1}^{\left(i\right)}}\right)}_{\left(j\right)},j=1,2\cdot \cdot \cdot ,n$$
  (14) $$\chi_{(j)k-1}^{\left(i\right)}={\bar{\chi }}_{k-1}^{\left(i\right)}+\gamma {\left(\sqrt{P_{k-1}^{\left(i\right)}}\right)}_{\left(j-n\right)},j=n+1,n+2\cdot \cdot \cdot ,2n$$
  where $\gamma =\sqrt{n+\lambda }$, $\lambda =\alpha^{2}(n+\kappa )-n$, $10^{-4}\leq \alpha \leq 1$, κ=3−n, n is status dimension;
  Time updating is:
  (15) $$\{\begin{array}{l} \chi_{k/k-1}^{\left(i\right)}=f(\chi_{k-1}^{\left(i\right)},u_{k-1})\\ {\bar{\chi }}_{k/k-1}^{\left(i\right)}=\sum_{j=0}^{2n}W_{j}^{\left(m\right)}\chi_{(j)k/k-1}^{\left(i\right)}\\ P_{k/k-1}^{\left(i\right)}=\sum_{j=0}^{2n}W_{j}^{\left(c\right)}(\chi_{(j)k/k-1}^{\left(i\right)}-\chi_{k/k-1}^{\left(i\right)}){\left(\chi_{(j)k/k-1}^{\left(i\right)}-\chi_{k/k-1}^{\left(i\right)}\right)}^{T}+Q_{k-1}\\ Z_{(j)k/k-1}^{\left(i\right)}=h(\chi_{(j)k/k-1}^{\left(i\right)})\\ {\bar{Z}}_{k/k-1}^{\left(i\right)}=\sum_{j=0}^{2n}W_{j}^{\left(m\right)}Z_{(j)k/k-1}^{\left(i\right)} \end{array}$$
  where:
  (16) $$W_{0}^{\left(m\right)}=\frac{\lambda }{n+\lambda }$$
  (17) $$W_{0}^{\left(c\right)}=\frac{\lambda }{n+\lambda }+1-\alpha^{2}+\beta$$
  (18) $$W_{j}^{\left(m\right)}=W_{j}^{\left(c\right)}=\frac{1}{2(n+\lambda )},j=1,2,\cdot \cdot \cdot ,2n$$
  $\lambda =\alpha^{2}(n+\kappa )-n$, $10^{-4}\leq \alpha \leq 1$, κ=3−n, and for the normal distribution, β=2;
  Measuring updating is:
  (19) $$\{\begin{array}{l} P_{(zz)k/k-1}^{\left(i\right)}=\sum_{j=0}^{2n}W_{j}^{\left(c\right)}(Z_{(j)k/k-1}^{\left(i\right)}-{\bar{Z}}_{k/k-1}^{\left(i\right)}){\left(Z_{(j)k/k-1}^{\left(i\right)}-{\bar{Z}}_{k/k-1}^{\left(i\right)}\right)}^{T}+R_{k}\\ P_{(xz)k/k-1}^{\left(i\right)}=\sum_{j=0}^{2n}W_{j}^{\left(c\right)}(\chi_{(j)k/k-1}^{\left(i\right)}-{\bar{\chi }}_{k/k-1}^{\left(i\right)}){\left(Z_{(j)k/k-1}^{\left(i\right)}-{\bar{Z}}_{k/k-1}^{\left(i\right)}\right)}^{T}\\ K_{k}^{\left(i\right)}=P_{(xz)k/k-1}^{\left(i\right)}{\left(P_{(zz)k/k-1}^{\left(i\right)}\right)}^{-1}\\ {\bar{\chi }}_{k}^{\left(i\right)}=\chi_{k/k-1}^{\left(i\right)}+K_{k}^{\left(i\right)}(Z_{k}-{\bar{Z}}_{k/k-1}^{\left(i\right)})\\ P_{k}^{\left(i\right)}=P_{k/k-1}^{\left(i\right)}-K_{k}^{\left(i\right)}P_{(zz)k/k-1}^{\left(i\right)}{\left(K_{k}^{\left(i\right)}\right)}^{T} \end{array}$$
  Using the particle $\chi_{k}^{\left(i\right)}$ generation according to the recommended density function $q(X_{k}^{\left(i\right)}/(X_{0}^{k}(i),Z_{0}^{k}))\approx N({\bar{\chi }}_{k}^{\left(i\right)},P_{k}^{\left(i\right)})$ as the second sampling original particles;
3. According to the weight value updating formula $w_{k}^{\left(i\right)}=w_{k-1}^{\left(i\right)}\frac{p(Z_{k}/\chi_{k}^{\left(i\right)})p(\chi_{k}^{\left(i\right)}/\chi_{k-1}^{\left(i\right)})}{q[\chi_{k}^{\left(i\right)}/(\chi_{0}^{k}(i),Z_{0}^{k})]}$, the corresponding weight values of N particles are calculated and the normalization processing is performed;
4. The original particles $\chi_{k}^{\left(i\right)}(i=1,2,\cdot \cdot \cdot ,N)$ are re-sampled by re-sampling algorithms to generate the second sampled particles $\chi_{k}^{\left(j\right)}(j=1,2,\cdot \cdot \cdot ,N)$ and their weight values are calculated;
5. The optimal estimation of state variables and the corresponding covariance matrix of each particle are calculated according to ${\hat{X}}_{k}=w_{k}^{\left(j\right)}\sum_{j=1}^{N}\chi_{k}^{\left(i\right)}$;
6. The particles $\chi_{k}^{\left(j\right)}$ after re-sampling in step (4) and ${\tilde{P}}_{k}^{\left(i\right)}$ calculated in step (5) are substituted in step (2) for the iterative calculation.

### 3.2. The Adaptive UPF Algorithm in This Paper

The time-varying gradually fading factors are used to weaken the influence that the obsolete data on the current filtering value based on the idea of strong tracking filter, the covariance of state prediction error, and the corresponding gain matrix are adjusted in real-time to achieve this purpose. To some extent, the adaptive UPF can judge the convergence of the system in real time and improve the correction of the current information error to the system filter by introducing memory attenuation factor, it can also slow down the degradation of UPF particle and accelerate the convergence rate of the particle filter.

The adaptive measure taken in this paper is to judge the covariance of the filter, the specific formula is as follows [14,15]:

(20) $${\tilde{Z}}_{k}^{T}{\tilde{Z}}_{k}\leq S*tr(E({\tilde{Z}}_{k}{\tilde{Z}}_{k}^{T}))$$

where S is the setting adjustment coefficients, and generally S > 1; ${\tilde{Z}}_{k}$ is the residual error array of the system, ${\tilde{Z}}_{k}=Z_{k}-h({\bar{X}}_{k|k-1})$.

When Equation (14) is not true, $P_{k/k-1}^{\left(i\right)}$ is needed to carry out the effective amendment, the approach in this paper is the introduction of the adaptive weighting coefficient $\lambda_{k}$ of the attenuation memory factors, the specific definition can be expressed as:

(21) $$\lambda_{k}=\{\begin{array}{l} \lambda_{0},\lambda_{0}\geq 1\\ 1,\lambda_{0}<1 \end{array}$$

The correction formula is:

(22) $$P_{k/k-1}^{\left(i\right)}=\lambda_{k}\sum_{j=0}^{2n}W_{j}^{\left(c\right)}(\chi_{(j)k/k-1}^{\left(i\right)}-{\bar{\chi }}_{k/k-1}^{\left(i\right)}){\left(\chi_{(j)k/k-1}^{\left(i\right)}-{\bar{\chi }}_{k/k-1}^{\left(i\right)}\right)}^{T}+Q_{k-1}$$

where:

(23) $$\lambda_{0}=\frac{tr(C_{0,k}-R)}{tr(\sum_{j=0}^{2n}W_{j}^{\left(c\right)}(\chi_{(j)k/k-1}^{\left(i\right)}-{\bar{\chi }}_{k/k-1}^{\left(i\right)}){\left(Z_{(j)k/k-1}^{\left(i\right)}-{\bar{Z}}_{k/k-1}^{\left(i\right)}\right)}^{T})}$$

(24) $$C_{0,k}=\{\begin{array}{l} {\tilde{Z}}_{k}{{\tilde{Z}}_{k}}^{T},k=1\\ \frac{\rho C_{0,k-1}+{\tilde{Z}}_{k}{{\tilde{Z}}_{k}}^{T}}{1+\rho },\;k>1 \end{array}$$

where 0<ρ≤1, its main role is to enhance quick tracking capability of the filter to the system state, the larger it is, the larger the assigned weight value of the current information is, the impact residual error of the current information on the estimate of the system is also more prominent. In order to ensure that the system has the ability of strong tracking with slowly changing circumstances and mutational status, ρ=0.95 in this paper.

In this paper, the implementation steps of the adaptive UPF are as follows:

1. Initialization: k = 0; we sample particles $\chi_{0}^{\left(i\right)}(i=1,2,3\cdot \cdot \cdot ,N)$ from the initial probability distribution $p(x_{0})$, for the simplified calculation, let $\chi_{0}^{\left(i\right)}\sim N({\bar{\chi }}_{0}^{\left(i\right)},P_{0}^{i})$, where ${\bar{\chi }}_{0}^{\left(i\right)}=\chi_{0}^{\left(i\right)}$, $P_{0}^{i}=P_{0}$;
2. Forecast updating:
  According to Equations (15) and (19), ${\bar{\chi }}_{k/k-1}^{\left(i\right)}$ and ${\bar{Z}}_{k/k-1}^{\left(i\right)}$ are obtained. Then the specific covariance is as follows:
  (25) $$P_{k/k-1}^{\left(i\right)}=\sum_{j=0}^{2n}W_{j}^{\left(c\right)}(\chi_{(j)k/k-1}^{\left(i\right)}-{\bar{\chi }}_{k/k-1}^{\left(i\right)}){\left(\chi_{(j)k/k-1}^{\left(i\right)}-{\bar{\chi }}_{k/k-1}^{\left(i\right)}\right)}^{T}+Q_{k-1}$$
3. Judge whether Equation (21) is satisfied or not; if satisfied, skip to the fifth step, otherwise correct $P_{k/k-1}^{\left(i\right)}$ in accordance with Equations (22), (23) and (24);
4. Measurement updating:
  (26) $${\bar{x}}_{k}^{\left(i\right)}={\bar{x}}_{k,k-1}^{\left(i\right)}+K_{k}(z_{k}-{\bar{z}}_{k,k-1}^{\left(i\right)})$$
  (27) $$P_{k}^{\left(i\right)}=P_{k,k-1}^{\left(i\right)}-K_{k}P_{zz}K_{k}^{T}$$
5. According to the weight value updating formula $w_{k}^{\left(i\right)}=w_{k-1}^{\left(i\right)}\frac{p(Z_{k}/\chi_{k}^{\left(i\right)})p(\chi_{k}^{\left(i\right)}/\chi_{k-1}^{\left(i\right)})}{q[\chi_{k}^{\left(i\right)}/(\chi_{0}^{k}(i),Z_{0}^{k})]}$, the corresponding weight values of N particles are calculated and normalized;
6. The original particles $\chi_{k}^{\left(i\right)}(i=1,2,\cdot \cdot \cdot ,N)$ are re-sampled by re-sampling algorithms to generate the second sampled particles $\chi_{k}^{\left(j\right)}(j=1,2,\cdot \cdot \cdot ,N)$ and their weight values are calculated;
7. The optimal estimation of state variables and corresponding covariance matrix of each particle are calculated according to ${\hat{X}}_{k}=w_{k}^{\left(j\right)}\sum_{i=1}^{N}\chi_{k}^{i}$;
8. The particles $\chi_{k}^{\left(j\right)}$ after re-sampling in the sixth step and ${\tilde{P}}_{k}^{\left(j\right)}$ calculated in the seventh step are substituted in the second step for the iterative calculation.

## 4. Adaptive UPF Filter Influence Factors Analysis

### 4.1. The Influence of the Importance Probability Density Function on the Accuracy of Adaptive UPF

The selection of the importance probability density function embodies in the weight updating section of particles, namely [16,17]:

(28) $$w_{k}^{\left(i\right)}=w_{k-1}^{\left(i\right)}\frac{p(Z_{k}/\chi_{k}^{\left(i\right)})p(\chi_{k}^{\left(i\right)}/\chi_{k-1}^{\left(i\right)})}{q[\chi_{k}^{\left(i\right)}/(\chi_{0}^{k}(i),Z_{0}^{k})]}$$

The ideal importance probability density function can ensure $Var(w_{k}^{j})=0$, namely the degradation of the particles can be completely eliminated. However, the optimum importance probability density function requires sampling from $p(\chi_{k}^{\left(i\right)}/\chi_{k-1}^{\left(i\right)},Z_{k})$, which is very difficult to carry out under the circumstance that the posterior probability density is a non-Gaussian case. In this paper, the specific method is:

(29) $$p(Z_{k}/\chi_{k}^{\left(i\right)})=e^{\left[-0.5*{\left(Z_{k}-Z_{k/k-1}\right)}^{T}*{\left(R\right)}^{-1}*(Z_{k}-Z_{k/k-1})\right]}$$

(30) $$p(\chi_{k}^{\left(i\right)}/\chi_{k-1}^{\left(i\right)})=e^{\left[-0.5*{\left(Z_{k}-Z_{k/k-1}\right)}^{T}*{\left(Q\right)}^{-1}*(Z_{k}-Z_{k/k-1})\right]}$$

(31) $$q(X_{k}^{\left(i\right)}/(X_{0}^{k}(i),Z_{0}^{k})={\left(\sqrt{\left|P_{k}\right|}\right)}^{-1}*e^{\left[-0.5*{\left(Z_{k}-Z_{k/k-1}\right)}^{T}*{\left(P_{k}\right)}^{-1}*(Z_{k}-Z_{k/k-1})\right]}$$

where $p(Z_{k}/\chi_{k}^{\left(i\right)}),\;p(\chi_{k}^{\left(i\right)}/\chi_{k-1}^{\left(i\right)}),\;q(X_{0}^{k}(i),Z_{0}^{k})$ can be modified according to the actual situation, but the modification is intended to achieve making the weight value of a particle smaller when the difference between the predicted particle and the actual status is larger, when the predicted particle is in good agreement with the actual status, the weight value of the particle should be larger.

### 4.2. Influence of Re-Sampling Algorithm on the Filtering Accuracy

Re-sampling is raised against the degradation issue of the particle weight value. Its purpose is to remove the particles with small weight value, increase the particles with large weight value, while the total number of particles is maintained constant. There are four kinds of commonly-used and representative re-sampling strategies, namely polynomial re-sampling, stratified re-sampling, system re-sampling, and residual error re-sampling [2,18]. Merwe *et al.* [18] pointed out that, regardless of the re-sampling method you choose, the impact on the PF calculation method is not very large [2]. In this paper, simulation is done to analyze three kinds of methods: system re-sampling, remainder re-sampling, and polynomial re-sampling.

## 5. Simulation and its Analysis

### 5.1. Simulation Conditions

Under the condition of static base, gyro constant drift is 0.01°/*h*, random drift is 0.001°/$\sqrt{h}$; accelerometer zero bias is 100 µg (g = 9.8 m^2^/s), random deviation is 50 µg; the local geographic latitude is 32.37°, longitude is 118.22°. Simulation time is 2000 s.

### 5.2. Simulation Results and Analysis

#### 5.2.1. The First Experiment

**Figure 1:**
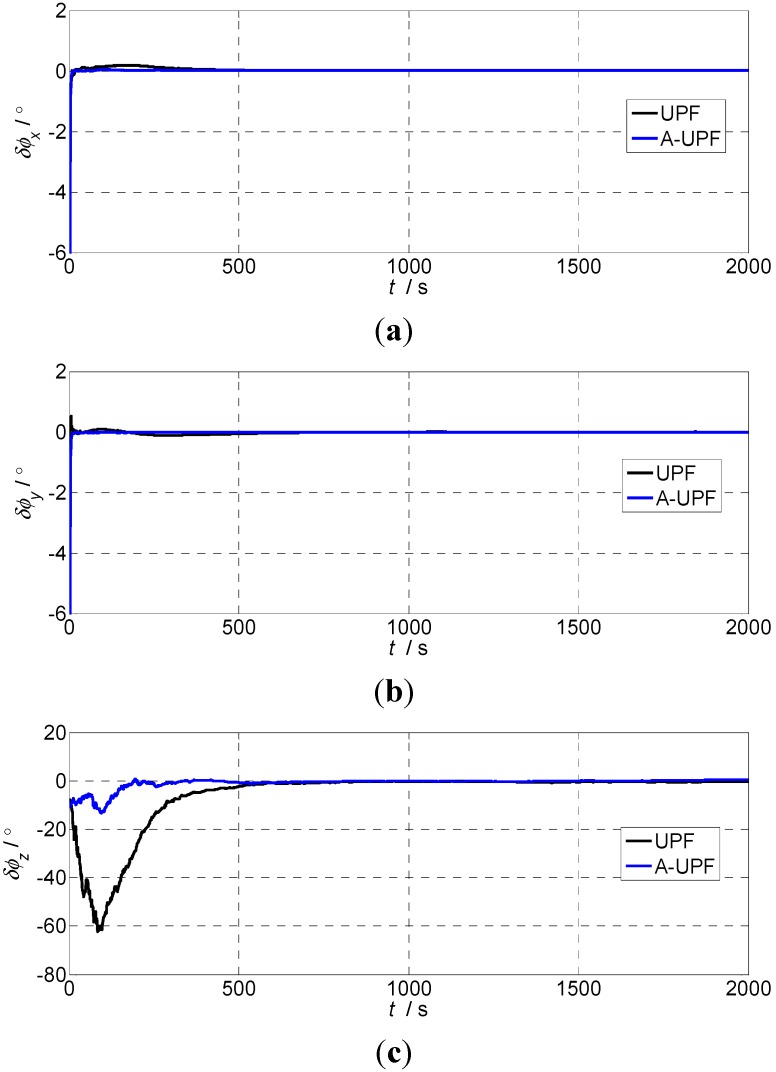
The alignment errors of large azimuth misalignment angle ($\varphi (0)={\left[\begin{array}{ccc} 1{}^{\circ} & 1{}^{\circ} & 10{}^{\circ} \end{array}\right]}^{T}$): (**a**) Pitch errors; (**b**) Roll errors; (**c**) Heading errors.

**Figure 2:**
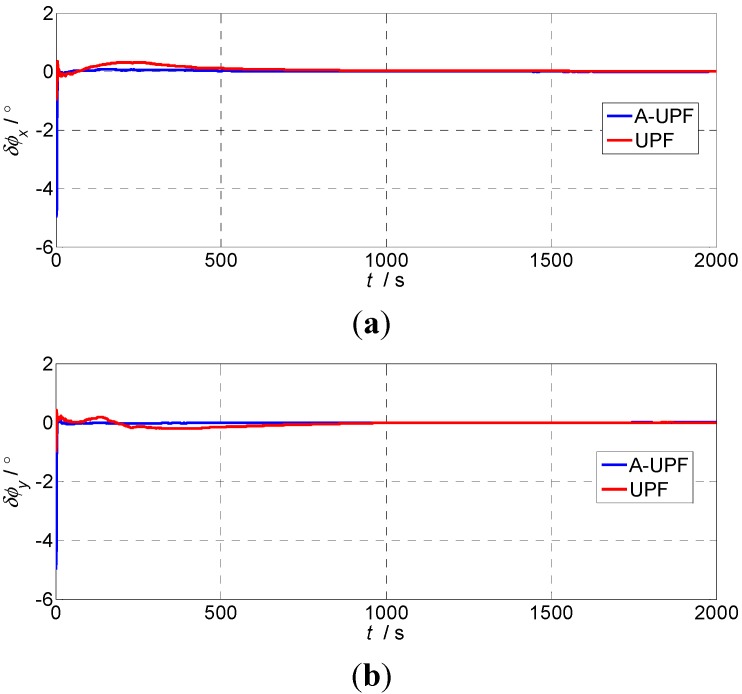

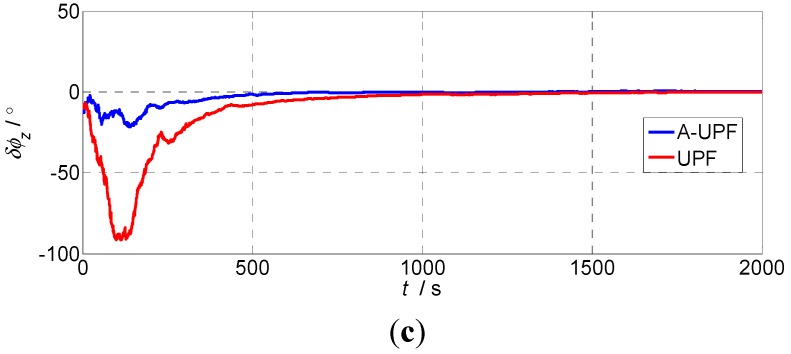
The alignment errors of large misalignment angles ($\varphi (0)={\left[\begin{array}{ccc} 1{}^{\circ} & 1{}^{\circ} & 10{}^{\circ} \end{array}\right]}^{T}$): (**a**) Pitch errors; (**b**) Roll errors; (**c**) Heading errors.

In accordance with the large azimuth misalignment angle error model, we partly use two kinds of filtering algorithms for the simulation experiment in the case where noise statistical properties are determined. Now we select the initial misalignment angle as $\varphi (0)={\left[\begin{array}{ccc} 1{}^{\circ} & 1{}^{\circ} & 10{}^{\circ} \end{array}\right]}^{T}$, the feedback correction is not performed during the simulation process in both cases; the simulation results of the alignment error are shown in Figure 1 and Figure 2.

**Table 1:** Determining the statistical properties of the noise are the statistical results of the alignment.

	Mean/°			Variance/°		
	Pitch Error	Roll Error	Heading Error	Pitch Error	Rollerror	Heading Error
UPF	0.0209	0.0189	1.0983	0.0524	0.0567	2.3819
Adaptive UPF	−0.0181	0.0074	−0.8609	0.0555	0.0423	1.3958

Figure 1 shows that when the initial misalignment angle is $\varphi (0)={\left[\begin{array}{ccc} 1{}^{\circ} & 1{}^{\circ} & 10{}^{\circ} \end{array}\right]}^{T}$, and we use the adaptive UPF, the horizontal alignment time required less than 50 s, the azimuth alignment time required less than 300 s; using UPF, the horizontal alignment time needs about 300 s, and the azimuth alignment time is about 500 s based on SINS error model with large azimuth misalignment angle. The alignment time of the adaptive UPF is obviously superior to UPF, but alignment accuracy of both is considerable. It can be seen that to some extent the alignment accuracy using adaptive UPF is higher than UPF from Table 1.

#### 5.2.2. The Second Experiment

In order to verify the filtering performance of the two kinds of filtering methods under the uncertain noise situation, we specifically add noise whose variances are all 0.02 to the acceleration of three directions to do experimental analysis. Take $\varphi (0)={\left[\begin{array}{ccc} 1{}^{\circ} & 1{}^{\circ} & 10{}^{\circ} \end{array}\right]}^{T}$. As it can be seen from Figure 2, when the measurement noise increases, the alignment of the horizontal direction will have a substantial shock after using UPF and the system may have been affected to some extent. The horizontal alignment time is about 700 s, the azimuth alignment time is about 900 s. After using the adaptive UPF, the level alignment time is significantly better than UPF, about 50 s, and the error curve is smooth. The azimuth alignment needs 450 s, so the alignment time is much shorter than UPF. From the statistical results of Table 2, the alignment accuracy using the adaptive UPF is significantly better than UPF.

**Table 2:** The statistical properties of uncertain noise are the statistical results of alignment.

	Mean/°			Variance/°		
	Pitch Error	Roll Error	Head Error	Pitch Error	Roll Error	Head Error
UPF	0.0454	0.0278	2.8174	0.0648	0.0634	4.9038
Adaptive UPF	0.0105	0.0122	−0.7304	0.0629	0.0425	2.2557

#### 5.2.3. The Third Experiment

**Figure 3:**
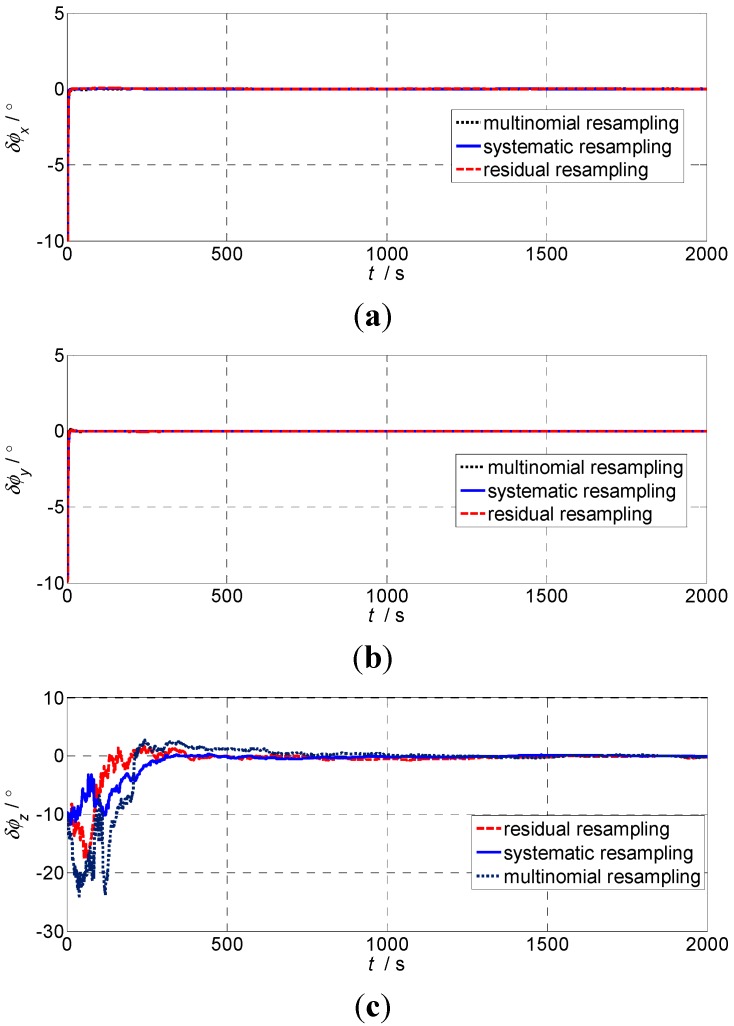
The influence of different re-sampling algorithms on alignment accuracy: (**a**) Pitch errors; (**b**) Roll errors; (**c**) Heading errors.

This paper also analyzes the influence of re-sampling algorithms on the filtering effect of the adaptive UPF by experiment. Figure 3 shows the results of residual re-sampling, the systematic re-sampling, multinomial re-sampling applied with the adaptive UPF.

Figure 3 shows the alignment accuracy of the three re-sampling algorithms is comparable after the alignment is finished but, initially, using a multinomial re-sampling algorithm has larger and longer time jitter in the horizontal and heading direction, so we can select a different re-sampling algorithm based on the system.

## 6. Turntable Experiment

The initial alignment of a large azimuth misalignment angle in SINS based on adaptive UPF proposed in this paper has been verified by a turntable experiment. The experiment was run as a semi-physical simulation with data collected from the turntable and SINS.

### 6.1. Experiment Setup

#### 6.1.1. Turntable and SINS

**Figure 4:**
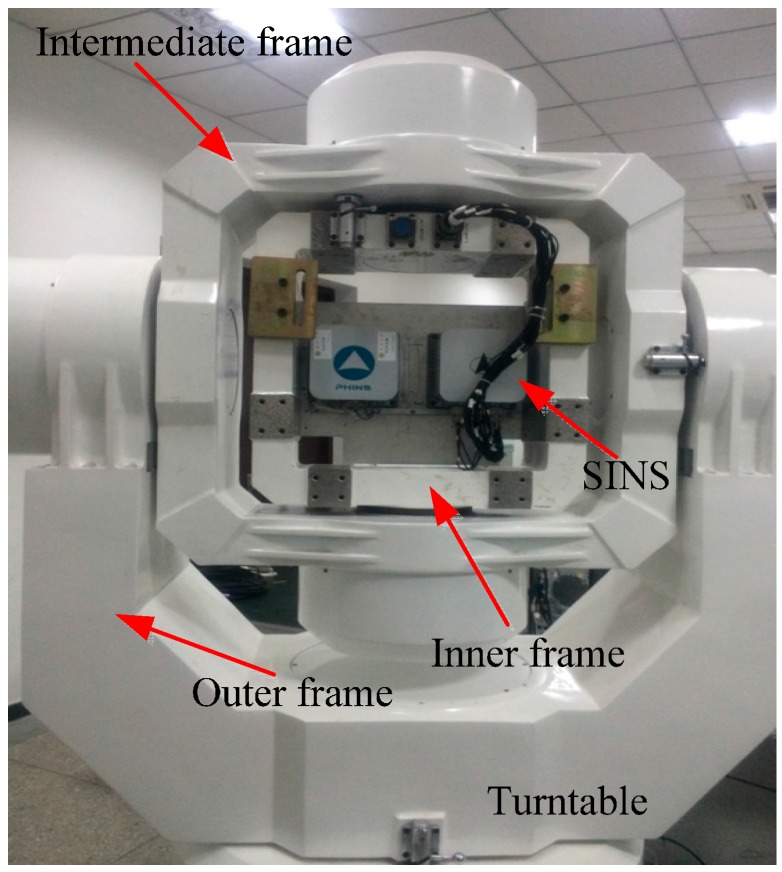
The turntable and SINS.

The turntable used in this experiment is shown in Figure 4. In the turntable, the rate controlling accuracy is ±0.0005°/s and angle measuring accuracy is ±0.0001°. In addition, angle information can be provided via a serial communication port as a response to the external time-synchronization signal. In the experiment, the inner, intermediate, and outer frames are used to simulate the ship’s roll, pitch, and yaw respectively.

The strapdown inertial navigation system used in this experiment which is developed by Casic33s is shown in Figure 4. Fiber optic gyros and quartz accelerometers are used in this type of SINS. The sensor precision of the SINS is provided in Table 3. The update frequency of the turntable data and SINS sensor data are 100 Hz.

**Table 3:** Sensor precision of SINS.

Gyro		Accelerometer	
Constant errors	0.006°/*h*	Constant errors	50 µg
Random errors	0.006°/$\sqrt{h}$	Random errors	50 µg

The constant drift, scale factors, cross coupling coefficient, installation error angle, and so on, can be calculated and compensated by the exact calibration according to [16,19], so these errors all can be ignored in the calibration.

#### 6.1.2. Construction of the Experimental Environment

**Figure 5:**
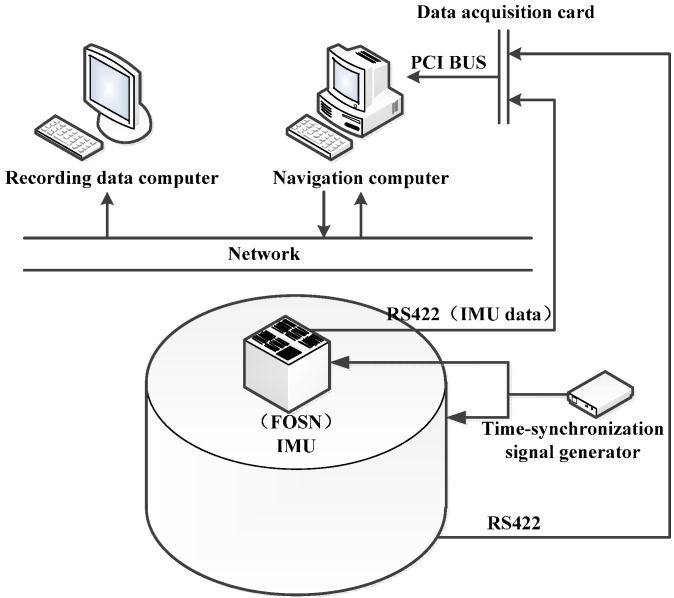
Experimental environment.

As shown in Figure 5, the experimental environment consists of a turntable, IMU (FOSN), computer, time-synchronization signal generator, data acquisition card, local area network, serial communication port, and so on.

A time-synchronization signal of 100 Hz is introduced to make the IMU data and turntable data synchronous. In this experimental environment, once the time-synchronization signal is active, the current angle information of the turntable and the information of the IMU will be sent to the navigation computer via the serial communication port, then the current sensor data will be collected for navigation solution and data fusion and, finally, navigation parameters will be sent back to the recording data computer at 1 Hz by the navigation computer. Additionally, data from the SINS and the turntable should be stored in the recording data computer in order to evaluate different alignment algorithms.

### 6.2. Experimental Results and Analysis

**Figure 6:**
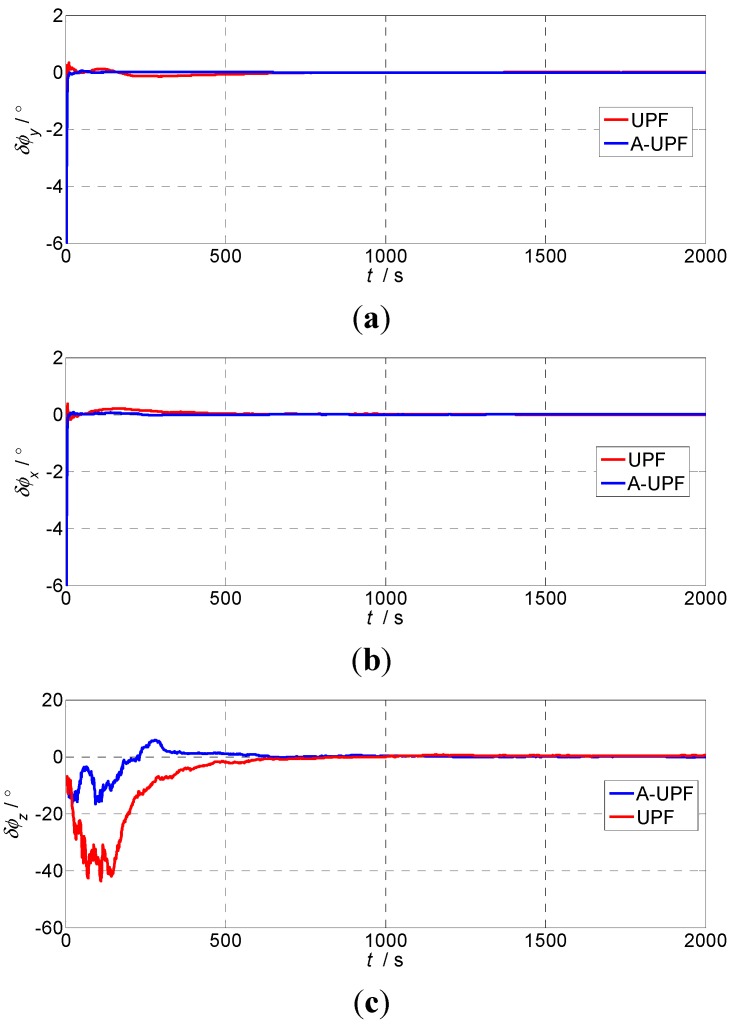
Estimation error curves for misalignment angles: (**a**) Pitch errors; (**b**) Roll errors; (**c**) Heading errors.

During the experiment, the inner and intermediate frames of the turntable are constantly kept in a level status. The outer frame rotates to 10° (this value can be selected randomly); then we can consider the carrier theoretical attitude value as ${\left[\begin{array}{ccc} 0{}^{\circ} & 0{}^{\circ} & 10{}^{\circ} \end{array}\right]}^{T}$. Two alignment schemes based on UPF and adaptive UPF are compared through the semi-physical simulation. The experiment lasts for 2000 s. Estimation error curves for the misalignment are shown as Figure 6.

From Figure 6, it can be seen that, when using the adaptive UPF, the horizontal alignment time requires less than 50 s, the azimuth alignment time requires about 500 s; using UPF, the horizontal alignment time needs about 300 s, and the azimuth alignment time is about 700 s, based on the SINS error model with a large azimuth misalignment angle. The alignment time of the adaptive UPF is obviously superior to UPF.

## 7. Conclusions

Based on the established nonlinear state equation of SINS with a large azimuth misalignment error, the initial alignment adopts UPF and the adaptive UPF under the circumstance of the large azimuth misalignment in SINS, where the statistical noise characteristics are fixed or not. The simulation shows that when the noise statistical properties are certainly determined, using the adaptive UPF has faster alignment speed than UPF, while the alignment accuracy advantage is not obvious. When the noise statistical properties are uncertainly determined, comparing the adaptive UPF with the normal UPF, the speed and the accuracy of alignment has obvious advantages. In addition, it is found that the system error caused by a residual error re-sampling algorithm is larger than the systematic re-sampling and polynomial re-sampling by choosing different re-sampling algorithms, which cannot be ignored in the high-precision navigation systems. Turntable experiments were done to certify the feasibility and superiority of the initial alignment of large azimuth misalignment angle in SINS base on adaptive UPF. They provide theoretical evidence and a calculation method for the initial alignment of large azimuth misalignment angles on a static base of SINS based on adaptive UPF in engineering, and at the same time provides a new idea for the initial alignment on a moving base of SINS based on adaptive UPF.
